# Expression of inhibitor of apoptosis protein Livin in renal cell carcinoma and non-tumorous adult kidney

**DOI:** 10.1038/sj.bjc.6604028

**Published:** 2007-10-30

**Authors:** N Wagener, I Crnković-Mertens, C Vetter, S Macher-Göppinger, J Bedke, E F Gröne, H Zentgraf, M Pritsch, K Hoppe-Seyler, S Buse, A Haferkamp, F Autschbach, M Hohenfellner, F Hoppe-Seyler

**Affiliations:** 1 German Cancer Research Center, Molecular Therapy of Virus-Associated Cancers (F065), Im Neuenheimer Feld 242, 69120 Heidelberg, Germany; 2 Department of Urology, University of Heidelberg, Im Neuenheimer Feld 110, 69120 Heidelberg, Germany; 3 Department of Pathology, University of Heidelberg, Im Neuenheimer Feld 220/221, 69120 Heidelberg, Germany; 4 German Cancer Research Center, Cellular and Molecular Pathology (E090), Im Neuenheimer Feld 280, 69120 Heidelberg, Germany; 5 German Cancer Research Center, Electron Microscopy (F090), Im Neuenheimer Feld 242, 69120 Heidelberg, Germany; 6 Department of Medical Biometry, University of Heidelberg, Im Neuenheimer Feld 305, 69120 Heidelberg, Germany

**Keywords:** inhibitor of apoptosis, Livin/ML-IAP/KIAP, renal cell carcinoma, tumour therapy

## Abstract

The antiapoptotic *Livin/ML-IAP* gene has recently gained much attention as a potential new target for cancer therapy. Reports indicating that *livin* is expressed almost exclusively in tumours, but not in the corresponding normal tissue, suggested that the targeted inhibition of *livin* may present a novel tumour-specific therapeutic strategy. Here, we compared the expression of *livin* in renal cell carcinoma and in non-tumorous adult kidney tissue by quantitative real-time reverse transcription-PCR, immunoblotting, and immunohistochemistry. We found that *livin* expression was significantly increased in tumours (*P*=0.0077), but was also clearly detectable in non-tumorous adult kidney. Transcripts encoding Livin isoforms *α* and *β* were found in both renal cell carcinoma and normal tissue, without obvious qualitative differences. Livin protein in renal cell carcinoma samples exhibited cytoplasmic and/or nuclear staining. In non-tumorous kidney tissue, Livin protein expression was only detectable in specific cell types and restricted to the cytoplasm. Thus, whereas the relative overexpression of *livin* in renal cell carcinoma indicates that it may still represent a therapeutic target to increase the apoptotic sensitivity of kidney cancer cells, this strategy is likely to be not tumour-specific.

The inhibitor of apoptosis protein (IAP) family encompasses structurally related proteins which may contribute to the development and therapeutic resistance of cancers (Salvesen and Duckett, 2002). Livin, alternatively termed ML-IAP or KIAP, is one of eight known human IAPs (Chang and Schimmer, 2007; Liu *et al*, 2007). Two splicing variants of Livin, Livin *α* and Livin *β*, have been identified which possess an identical amino-acid sequence, except for additional 18 amino acids present only in the *α*-isoform (Vucic *et al*, 2000; Ashhab *et al*, 2001). It has been shown that targeted interference with *livin* expression can resensitise tumour cells towards apoptosis (Kasof and Gomes, 2001; Crnković-Mertens *et al*, 2003).

Initially, a role for Livin in tumorigenesis had been proposed for malignant melanoma (Vucic *et al*, 2000). More recently, however, *livin* expression was also detected in various additional cancers, including leukaemias (Qiuping *et al*, 2004; Choi *et al*, 2007), bladder cancer (Gazzaniga *et al*, 2003), lung cancer (Tanabe *et al*, 2004; Hariu *et al*, 2005; Crnković-Mertens *et al*, 2006b), neuroblastoma (Kim *et al*, 2005), nasopharyngeal carcinoma (Xiang *et al*, 2006), astrocytoma (Liu *et al*, 2006), malignant pleural mesothelioma (Gordon *et al*, 2007), pancreatic cancer (Lopes *et al*, 2007), and renal cell carcinoma (RCC) (Crnković-Mertens *et al*, 2007). Notably, studies of biopsies from bladder cancer (Gazzaniga *et al*, 2003), lung cancer (Hariu *et al*, 2005), nasopharyngeal carcinoma (Xiang *et al*, 2006), malignant pleural mesothelioma (Gordon *et al*, 2007), and pancreatic cancer (Lopes *et al*, 2007) reported that Livin is typically expressed in the tumour samples, but not in the corresponding normal tissues. Thus, it could be envisioned to develop the targeted inhibition of *livin* as a future tumour-specific therapeutic strategy (Chang and Schimmer, 2007; Liu *et al*, 2007). It therefore will be of great interest to determine the Livin status of different tumour entities in relation to the corresponding normal tissue.

Renal cell carcinoma (RCC) is estimated to account for more than 51 000 new cases and almost 13 000 cancer-related deaths in the United States in the year 2007, making it the second most lethal of the urological cancers (Jemal *et al*, 2007). Renal cell carcinomas typically are highly resistant towards chemotherapy and possess a poor prognosis (Flanigan *et al*, 2003). We recently found that silencing of Livin-positive RCC cell lines was linked to a re-sensitisation of the tumour cells towards proapoptotic agents, including chemotherapeutics (Crnković-Mertens *et al*, 2007). To evaluate the potential of *livin* to serve as a tumour-specific therapeutic target, we here compared its expression in primary RCC specimens and in non-tumorous tissue samples from adult kidney. In addition, we analysed the expression of *livin* isoforms *α* and *β* in tumorous and non-tumorous tissue.

## MATERIALS AND METHODS

### Tissue samples

Fresh-frozen tissue samples of RCC (*n*=15), macroscopically and histologically normal tissue derived from kidneys removed because of malignant disease (*n*=18), and tissue from kidneys removed because of benign disease (*n*=6) were obtained from the tissue bank of the National Center for Tumor Diseases (NCT) Heidelberg. These tissues included nine paired samples of tumour and adjacent non-tumorous tissue.

Paraffin-embedded tissues encompassed RCC samples (*n*=13), normal tissue adjacent to tumour (*n*=14) and kidney removed because of benign disease (*n*=3).

Tumour stage was classified according to the tumour node metastasis staging system (Greene *et al*, 2002). The work was covered by a votum of the Ethical Committee of the University of Heidelberg no. 206/2005. Written consent was obtained from each patient.

### RNA extraction and quantitative real-time reverse transcription (RT)–PCR

RNA extraction and qRT–PCR analyses were performed as described previously (Crnković-Mertens *et al*, 2006b). In brief, RNA was isolated from homogenised tissues by phenol–chloroform extraction. We also included a commercially available RNA sample, representing a mixture of 14 different RNAs derived from normal kidney, unrelated to cancer (Clontech, Mountain View, CA, USA). Reverse transcription of 1 *μ*g RNA was performed using the oligo-dT primer and SuperScriptIII First-Strand kit (Invitrogen, Karlsruhe, Germany). *Livin*, *glyceraldehyde-3-phosphate dehydrogenase* (*GAPDH*), and *hypoxanthine phosphoribosyl-transferase 1* (*HPRT1*) expression were determined by real-time PCR. Relative quantification was performed using the comparative *C*_t_ (2^−ΔΔ*C*_t_^) method (Livak and Schmittgen, 2001). Data are presented as the fold difference in gene expression normalised to a housekeeping gene index (the geometric mean of the expression levels of *GAPDH* and *HPRT1*), as an endogenous reference, and relative to a calibrator sample.

### Reverse transcription–PCR

Expression of *livin α* and *β* mRNAs was analysed by RT–PCR, using primers which distinguish between the two splice variants (Crnković-Mertens *et al*, 2006a). PCR products (153 bp for *livin α*, 99 bp for *livin β*) were analysed by agarose gel electrophoresis.

### Protein extraction from fresh-frozen tissue and Western blot analyses

Proteins were extracted from tumorous and non-tumorous adult kidney specimens, as described previously (Crnković-Mertens *et al*, 2007). Briefly, tissue sections were homogenised in lysis buffer, supplemented with protease inhibitors and Pefabloc (Biomol, Hamburg, Germany). Lysates were centrifuged at 100 000 **g** for 30 min at 4°C and supernatants collected. Proteins were separated by 12.5% SDS–polyacrylamide gel electrophoresis, transferred on to an Immobilon-P membrane (Millipore, Billerica, MA, USA), and detected with a monoclonal anti-Tubulin antibody (Oncogene, Boston, MA, USA). The hybridoma producing the monoclonal anti-Livin antibody no. 6 was raised in a male Balb/c mouse which was immunised by injection of purified Livin–hexa–His fusion protein. Western blots were performed by employing enhanced chemiluminescence (Millipore).

### Immunohistochemistry

Paraffin-embedded tissue samples were cut at 2 *μ*m, placed on slides, and dried for 24 h at 37°C. Sections were dewaxed, rehydrated using xylol and descending series of ethanol, and immersed in 3% H_2_O_2_ for 5 min to block endogenic peroxidases. After washing with TBST (50 mM Tris, 300 mM NaCl pH 7.6, 0.1% Tween), unspecific antibody binding sites were blocked using protein block solution provided by the Catalyzed Signal Amplification (CSA) II System (DAKO, Carpinteria, CA, USA). Sections were incubated for 30 min at room temperature with a monoclonal anti-human Livin mouse antibody (Active Motif, San Diego, CA, USA) at a dilution of 1 : 400. Sections were immersed in horseradish peroxidase-conjugated anti-mouse antibody for 15 min at room temperature, followed by an incubation with fluorescyl-tyramide hydrogen peroxide, for 15 min to intensify staining. Thereafter, sections were incubated with anti-fluorescein antibody conjugated to horseradish peroxidase and exposed to DAB solution (3,3′-diaminobenzidine tetrahydrochloride) for 2 min. Counterstaining of cell nuclei was carried out by immersing the section in hemalaun. Sections were thoroughly washed, glass covered, and analysed by light microscopy (Olympus Vanox-T, Hamburg, Germany), using a magnification of up to × 400. Tissue specimens were examined at random order by two independent examiners for the absence or presence of Livin staining in the cytoplasm or nucleus of tumorous and non-tumorous tissues.

### Statistics

*Livin* mRNA measurements were log transformed to achieve data, which can be assumed to be normally distributed. To compare the distributions of log-*livin* between tumour and non-tumour tissues, a mixed linear model with the patient as random factor was applied, to account for paired data in nine patients. The test for the difference in log-*livin* between the different tissues is two-sided with a significance level of *P*=0.05. Test analysis was carried out with the Statistical Analysis System, Version 9.1 for Windows (SAS Institute Inc., Cary, NC, USA).

## RESULTS

### Comparative analysis of Livin expression in RCCs and non-tumorous tissue

**Figure 1:**
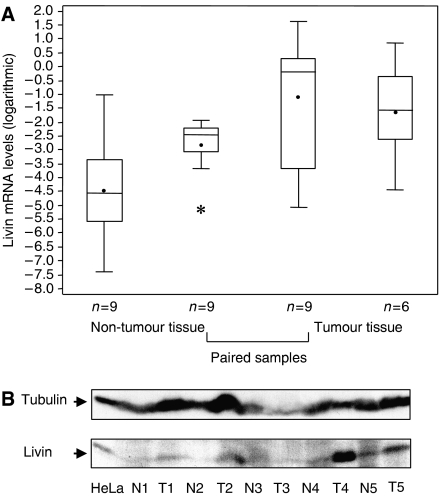
Livin mRNA and protein levels in renal cell carcinoma (RCC) tumour tissue and non-tumorous adult kidney. (**A**) *Livin* mRNA expression levels were measured by qRT–PCR in tissue specimens from RCC (*n*=15) and non-tumorous kidney tissue adjacent to tumour (*n*=18). This analysis included nine paired samples of tumour and corresponding non-tumorous tissue. Values were log transformed. A mixed linear model with the patient as a random factor was applied to account for paired data in nine patients when comparing the distributions of log-*livin* between different tissues. Log-*livin* measurements were visualised in box plots with upper whiskers drawn up to the maximum value below third quartile+1.5^*^ (interquartile range), lower whiskers defined accordingly, asterisk: outlier. (**B**) Western blot analysis of Livin protein in five paired samples in primary RCCs (T1–5) and non-tumorous tissue adjacent to the tumour (N1–5). HeLa cells served as positive control for Livin expression; Tubulin: loading control.

Quantitative real-time RT–PCR analyses were performed, to measure expression of the *livin* gene in (i) RCC tumour tissues, (ii) macroscopically and histologically normal tissue derived from kidneys removed because of malignant disease, and (iii) tissue from kidneys removed because of benign disease. *Livin* transcripts were detectable in all specimens examined. We found that *livin* mRNA levels in tumour tissue were significantly higher (*P*=0.0077) than in samples from normal adult kidney tissue (Figure 1A). *Livin* transcript levels in normal tissue and in specimens from benign kidney diseases were in the same range as was a commercially available sample representing a mixture of 14 RNAs derived from normal kidney (data not shown).

To investigate whether the difference in Livin expression between tumorous and non-tumorous samples is reflected at the protein level, we analysed five paired samples (tumour tissue and adjacent normal tissue from the same patient) by direct protein extraction from the tissues. As shown in Figure 1B, Livin protein levels were clearly detectable in all tumour samples, but close to the detection limit of this method in adjacent normal tissue. Taken together, these findings demonstrate that both the *livin* gene and the Livin protein are expressed in tumorous and non-tumorous kidney tissues, with significantly increased expression in RCCs.

### Analysis of Livin isoforms *α* and *β* in RCCs and non-tumorous tissue

**Figure 2:**
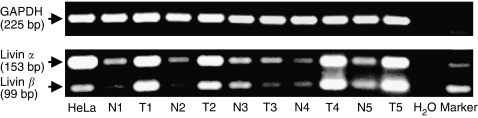
Analysis of primary renal cell carcinomas (RCCs) and non-tumorous adult kidney for the expression of mRNAs encoding Livin isoforms *α* and *β*. Five paired samples of tumour (T1–5) and adjacent non-tumorous tissue (N1–5) were analysed by isoform-specific RT–PCR. HeLa cells, which express both isoforms (Crnković-Mertens *et al*, 2006a), served as a positive control. *GAPDH*, internal standard.

We analysed expression of Livin isoforms *α* and *β* by isoform-specific RT–PCR (Crnković-Mertens *et al*, 2006a). Both *livin α* and *β* mRNA were consistently detected. Overall, *livin α* and *β* mRNAs expression levels were similar in 3 out of 9 tumour tissues and in 6 out of 13 non-tumorous adult kidney samples whereas 6 out of 9 tumours and 7 out of 13 non-tumorous adult kidney samples exhibited higher *livin α* mRNA levels. Thus, whereas the relative ratio between *livin α* and *β* varied for individual sample pairs, it did not show an obvious link to tumorigenicity. Exemplary results for five corresponding samples from non-tumorous and tumorous tissue are shown in Figure 2.

### Immunohistochemical analysis of Livin protein expression in RCCs and non-tumorous tissue

**Figure 3:**
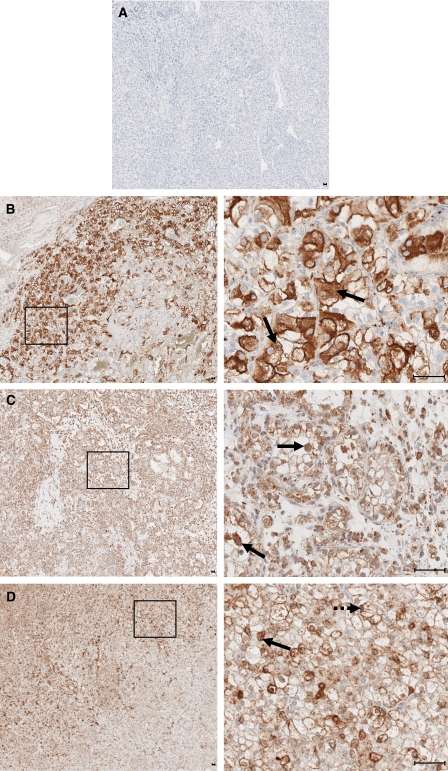
Immunohistochemical analysis of Livin protein expression in primary renal cell carcinomas (RCCs). (**A**) Negative control: RCC tissue specimen, incubated with mouse IgG instead of the anti-Livin antibody. (**B**) Overview (left panel) and higher resolution (right panel) of an RCC sample showing cytoplasmic localisation of Livin (arrows), sparing the nucleus. (**C**) Overview (left panel) and higher resolution (right panel) of an RCC sample showing nuclear localisation of Livin (arrows). (**D**) Overview (left panel) and higher resolution of an RCC sample exhibiting both nuclear (interrupted arrow) and cytoplasmic (arrow) staining. Higher resolved areas in the right panels correspond to the framed regions in the left panels, bars, 5 *μ*m.

**Figure 4:**
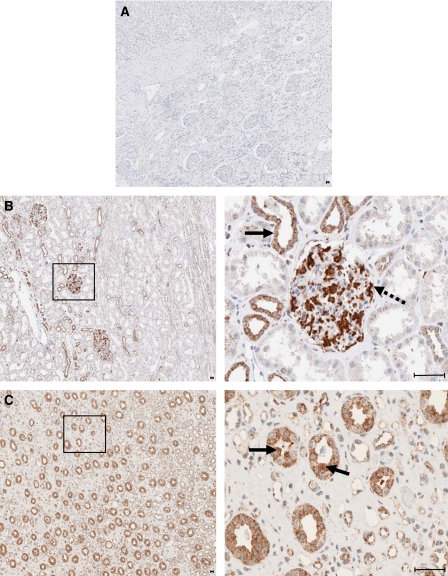
Immunohistochemical analysis of Livin protein expression in non-tumorous kidney. (**A**) Negative control: non-tumorous kidney, incubated with mouse IgG. (**B**) Overview (left panel) and higher resolution (right panel) of normal kidney, showing cytoplasmic localisation of Livin in distal tubule epithelial cells (arrow) and in glomerular mesangial cells and podocytes (interrupted arrow). (**C**) Overview (left panel) and higher resolution (right panel) of normal kidney, showing cytoplasmic localisation of Livin in distal tubule epithelial cells (arrows). Higher resolved areas in the right panels correspond to the framed regions in the left panels, bars, 5 *μ*m.

Next, we investigated the expression of Livin protein in paraffin-embedded tissues from RCCs and non-tumorous tissue, by immunohistochemistry. Livin expression was detectable in 10 out of 13 RCC tissue samples (Figure 3B–D). Moreover, Livin could be clearly visualised in all 17 specimens from non-tumorous adult kidney (Figure 4B and C). Livin expression was restricted to specific cells in non-tumorous kidney, namely to glomerular mesangial cells and podocytes (Figure 4B) as well as to distal and collect tubule epithelial cells (Figure 4B and C). Proximal tubular cells, which are thought to be the origin of most RCCs (Campbell *et al*, 2007), did not express detectable levels of Livin.

At the cellular level, Livin protein expression was detected in the cytoplasm of renal cancer cells in 7 out of 10 positive tumour specimens (Figure 3B). In addition, 6 out of 10 positive tumour specimens exhibited nuclear staining for Livin, to varying degrees (Figure 3C), which included three specimens staining positive both in the cytoplasm and nucleus (Figure 3D). In these latter cases, the ratio of cells with nuclear or cytoplasmic staining was approximately 30–70%. On the contrary, Livin expression in non-tumorous adult kidney cells was always limited to the cytoplasm (Figure 4B and C).

Taken together, these findings demonstrate that Livin is not only expressed in a large proportion of RCCs but also to a significant degree in specific cells of the normal kidney.

## DISCUSSION

In contrast to many other ubiquitously expressed IAPs, Livin is commonly considered to exhibit a largely tumour-specific expression pattern. This, together with the documented antiapoptotic activity of Livin, has raised considerable interest in developing strategies for the therapeutic inhibition of Livin in cancers (Chang and Schimmer, 2007; Liu *et al*, 2007). Conceptionally, these approaches would aim at correcting the increased apoptotic resistance of Livin-expressing cancer cells, thereby specifically increasing the susceptibility of tumour cells towards proapoptotic anticancer agents. Indeed, previous studies have shown that the targeted inhibition of *livin* by antisense oligonucleotides or RNA interference reduces the growth of Livin-expressing tumour cells in clonogenic survival assays and can resensitise them towards proapoptotic agents, including chemotherapeutics (Kasof and Gomes, 2001; Crnković-Mertens *et al*, 2003, 2006a, 2006b, 2007). Currently followed strategies aiming at the targeted inhibition of Livin include inhibitory peptides and small molecules (Franklin *et al*, 2003) and nucleic acid-based approaches (Crnković-Mertens *et al*, 2003). Since the specificity of these approaches would greatly profit from a tumour-specific expression of their target, we here investigated the expression of Livin in RCC and non-tumorous adult kidney.

Here, we demonstrate that Livin is not only expressed in RCCs, but also clearly detectable in specific cells of normal kidney. These findings contradict the common view that Livin expression is largely tumour-specific. Notably, previous studies did not detect *livin* expression in adult kidney by Northern blot analysis (Lin *et al*, 2000; Vucic *et al*, 2000; Kasof and Gomes, 2001), possibly due to the detection limit of this method. Only one RT–PCR analysis reported weak expression of *livin β*, but not *livin α* mRNA, in a single sample from adult kidney present in a commercially available multiple tissue cDNA panel (Ashhab *et al*, 2001). This finding was not followed further and is in contrast to our findings which indicate expression of both isoforms in non-tumorous tissue.

In non-tumorous kidney, highest levels of Livin protein were detected in glomerular mesangial cells and podocytes as well in distal and collecting tubule epithelial cells. These cells are in direct contact with urine during the filtration process. Although at present purely speculative, one could envision that antiapoptotic factors play a role for the protection of normal kidney cells from toxic substances in the urine.

It is remarkable that proximal tubular cells, from which most RCCs are believed to originate (Campbell *et al*, 2007), did not stain positive for Livin. Although we cannot exclude that these cells express low levels of Livin which are below the detection threshold of immunohistochemistry, the strong signals obtained in RCC cells indicate that an increase in Livin expression is acquired during RCC tumorigenesis. Furthermore, and in contrast to non-tumorous kidney tissue where Livin protein expression was restricted to the cytoplasm, we observed in 6 out of 10 positive tumour tissues nuclear staining. This suggests that Livin may also play a nuclear role during the transformation process. Nuclear activities which could be relevant for cell transformation have been demonstrated for other IAPs, such as survivin (Altieri, 2006) or cIAP1 (Samuel *et al*, 2005). Moreover, nuclear staining for IAPs has been related to the clinical prognosis of some tumours (Imoto *et al*, 2002; Li *et al*, 2005; Gordon *et al*, 2007). It therefore will be interesting to relate the subcellular localisation of Livin to the clinical prognosis of RCC patients in future studies, which will require higher patient numbers for statistical validation.

The significant levels of Livin expression in specific cells of normal kidney suggest that its targeted inhibition will not be a strictly tumour-specific therapeutic strategy. This should be taken into account for the development of *livin* inhibitors, to avoid unwanted side effects, such as nephrotoxicity. Furthermore, Livin has been reported to be a suitable target for the immunotherapy of lung cancer and of malignant melanoma where it can serve as a tumour rejection antigen (Schmollinger *et al*, 2003; Andersen *et al*, 2004; Schmollinger and Dranoff, 2004; Hariu *et al*, 2005). These immunotherapeutic strategies may also be complicated by expression of the targeted antigen in normal cells. Yet, we also would like to stress the point that our findings do not disqualify Livin as a therapeutic target. The observed overexpression of *livin* in RCCs may still allow a preferential, rather than a specific attack on tumour cells, depending on the therapeutic index of *livin* inhibitors. Accordingly, other IAPs overexpressed in cancers, such as XIAP or survivin, are considered as potential therapeutic targets, despite they are also expressed in normal tissues (Fukuda and Pelus, 2006; LaCasse *et al*, 2006).
